# Correlation between sagittal balance and thoracolumbar elastic energy parameters in 42 spines subject to spondylolisthesis or spinal stenosis and 21 normal spines

**DOI:** 10.1016/j.heliyon.2024.e38469

**Published:** 2024-09-26

**Authors:** Špela Bračun, Anna Romolo, Veronika Rehakova, Jure Leban, Žan Pukšič, Rok Vengust, Matej Daniel, Veronika Kralj-Iglič, Mitja Drab

**Affiliations:** a Surgical Centre Rožna Dolina, Rožna dolina cesta IV/45, SI-1000, Ljubljana, Slovenia; b Institution for Higher Education for Physiotherapy Fizioterapevtika, Slovenska cesta 58, SI-1000, Ljubljana, Slovenia; c University of Ljubljana, Faculty of Health Sciences, Laboratory of Clinical Biophysics, Zdravstvena 5, SI-1000, Ljubljana, Slovenia; d Department of Mechanics, Biomechanics and Mechatronics, Faculty of Mechanical Engineering, Czech Technical University in Prague, Technicka 4, CZ166-07 Prague 6, Czech Republic; e Department of Orthopaedic Surgery, University Medical Centre Ljubljana, Zaloška 9, SI-1000, Ljubljana, Slovenia; f University of Ljubljana, Faculty of Medicine, Vrazov trg 2, SI-1000, Ljubljana, Slovenia; g University of Ljubljana, Faculty of Electrical Engineering, Laboratory of Physics, Tržaška 25, SI-1000, Ljubljana, Slovenia

**Keywords:** Lumbar lordosis, Thoracolumbar spine, Elastic energy, Sagittal balance, Spine curvature

## Abstract

The curvature of the lumbar spine plays a critical role in maintaining spinal function, stability, weight distribution, and load transfer. We have developed a mathematical model of the lumbar spine curve by introducing a novel mechanism: minimization of the elastic bending energy of the spine with respect to two biomechanical parameters: dimensionless lumbosacral spinal curvature *c*_LS_ and dimensionless curvature increment along the spine CI. While most of the biomechanical studies focus on a particular segment of the spine, the distinction of the presented model is that it describes the shape of the thoracolumbar spine by considering it as a whole (non-locally) and thus includes interactions between the different spinal levels in a holistic approach. From radiographs, we have assessed standard geometrical parameters: lumbar lordosis LL, pelvic incidence PI, pelvic tilt PT, sacral slope ψ_0_ and sagittal balance parameter SB = sagittal vertical axis (SVA)/sacrum-bicoxofemoral distance (SFD) of 42 patients with lumbar spinal stenosis (SS) or degenerative spondylolisthesis (SL) and 21 radiologically normal subjects. SB statistically significantly correlated with model parameters *c*_L5_ (r = −0.34, p = 0.009) and −CI (r = 0.33, p = 0.012) but not with standard geometrical parameters. A statistically significant difference with sufficient statistical power between the patients and the normal groups was obtained for *c*_LS_, CI, and SB but not for standard geometrical parameters. The model provides a possibility to predict changes in the thoracolumbar spine shape in surgery planning and in assessment of different spine pathologies.

## 1 Introduction

The aging process and various spinal diseases lead to degenerative changes in the spine structure, resulting in alterations in its local geometry and biomechanics [[1], [2], [3], [4], [5], [6], [7]]. Such changes often induce also changes in parameters of sagittal balance - (physiological alignment of the spine in the most efficient manner [[8], [9], [10], [11], [12], [13]]) and contribute to chronic low back pain [14] and neck pain [15]. A precise quantitative analysis of spinal curvatures serves as a valuable tool in clinics and for the improvement of the understanding of how degenerative spinal disorders affect sagittal balance functions. Moreover, it assists in planning corrective surgical procedures for spine deformities [16,17].

Over the last century, researchers have developed various geometric and mathematical models to quantify the sagittal shape of the spine in two-dimensional radiographs, aiming to describe its morphology with only a few parameters. Various methods, from the modified Cobb approach [18] to numerical spline approximations [11], possess distinctive accuracies and reliabilities, detailed in a review by Vrtovec et al. (2009) [19]. The clinical utility of these models also hinges on the level of automation inherent in each approach. While the Cobb method, reliant on manual identification of end vertebrae, shows relatively high variability and unreliability, methods like the best-fit ellipses [20,21] already incorporate computerized image processing techniques such as edge detection and filtering. However, these methods do not provide the fundamental physical principles underlying the observed morphology. In clinical practice, also simpler methods focusing on the identification of a small number of relevant parameters are essential to enable physicians to address crucial issues in managing spine disorders.

Conceptualizing the spine in the sagittal plane as a linear chain linking the sacral plane to the head reveals the close mutual relationship between the shape and orientation of each segment of the spine relative to its neighboring segments [22]. Any alteration in shape or orientation at one level directly impacts adjacent segments. Upper body gravity, muscle forces, and soft tissues act as constraints and stabilize the spine according to the principle of minimal energy expenditure. This interconnectivity becomes evident in clinical scenarios involving surgical instrumentation. As local pathology can lead to global deformity, surgical treatment should consider regional features and global spinal alignment [23]. It was observed that after posterior cervical surgery due to cervical spondylosis, patients were more likely to lose lordosis of the cervical spine; it was suggested that this is caused by damage to the posterior structures and para-spinal muscles [24,25]. After cervical surgery, a weak correlation was found between lumbar degeneration and the change in cervical lordosis [26]. It was suggested that the loss of lordosis and occurrence of kyphosis after the surgery increased the mechanical stress in the front of the cervical spinal cord, which resulted in poor clinical outcomes [26]. Spinal trauma (e.g. compression fractures leading to kyphotic vertebral body deformity) can cause chronic changes in spinal balance [27]. Pedicle (two cylinder-shaped projections of hard bone that stick out from the back part of the vertebral body) screws used for correcting deformities not only affect the morphology of the instrumented levels but also influence overall posture [28]. These studies demonstrate that even a simple one-level lumbar instrumentation significantly influences sagittal parameters across the entire spine and can impact midterm clinical outcomes. Mathematically, local intrusions that disrupt the continuity of the spine's contour derivative at a specific point can significantly affect the new balance of the spine and impact regions beyond their immediate vicinity.

The spine functions as a connected entity, akin to a continuous curve. However, this approach, although well understood in clinical practice, has been largely neglected in biomechanical studies. Current studies focus either on a purely geometrical description of the spine shape, neglecting the role of mechanical components - or on a complex analysis of individual mechanical segments, neglecting the overall shape of the spine.

**Fig. 1:**
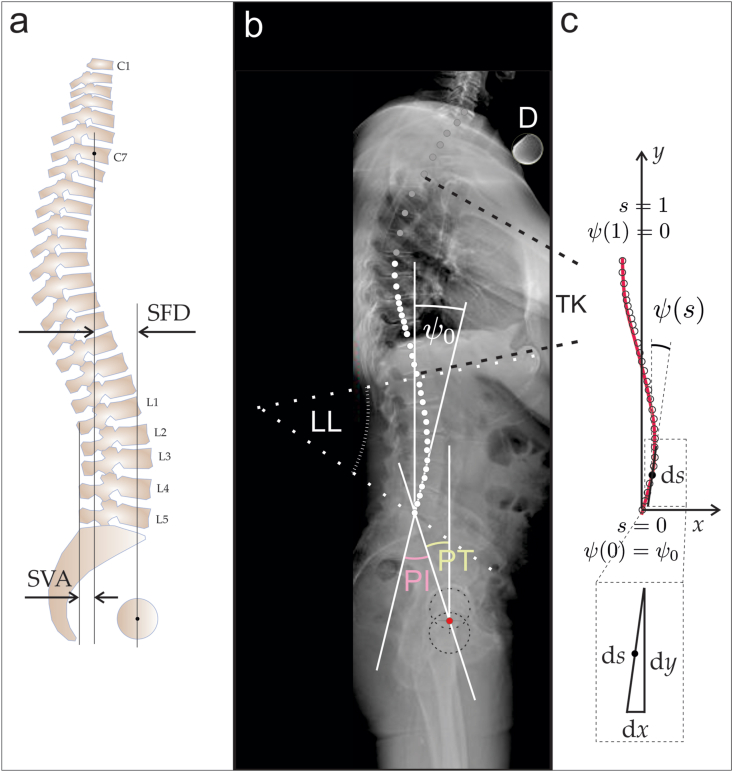
a: Scheme of the spine with parameters SVA and SFD which determine Sagittal Balance SB = SVA/SFD; C7 marks the 7th cervical vertebra and L1-L5 mark the lumbar vertebrae; **b:** A side radiograph of the spine in a two-legged stance with geometrical parameters: lumbar lordosis (LL), pelvic incidence (PI), pelvic tilt (PT) and sacral slope ψ_0_; D indicates a unit sphere with known diameter and TK indicates Thoracic Kyphosis; **c:** Shape of the spine assessed from an example radiograph (white dots), theoretically predicted shape of the spine (red line), and parametrization of the spine model curve by two dimensionless coordinates (*x* and *y*).

To emphasize the interconnection of the spine elements into one entity, we present a holistic model of the thoracolumbar spinal column in the sagittal plane based on the principle of its global energy minimization. This model does not take the shape of the spine as an input, but the thoracolumbar shape is predicted by minimizing the bending energy of the continuous spine curve while adhering to specified boundary geometric constraints. We seek the correlations of the model parameters with geometrical parameters of the spine and correlations of all (the geometrical and the biomechanical) parameters with sagittal balance parameter SB = SVA/SFD, where SVA is the sagittal vertical axis through the edge of the sacrum and SFD is the sacrum-bicoxofemoral distance in the horizontal direction (Fig. 1).

## 2 The model

The shape of the thoracolumbar spine is described as a two-dimensional curve lying in the sagittal plane of the body (vertical plane which passes through the body longitudinally). The global coordinate system was adopted with the coordinate *x*-axis pointing anteriorly and *y* being the vertical plumb line running through the middle of the sacral plane (Fig. 1a). The model is presented in the form of model equations (1)–(16) and additionally explained in Appendix 2 (including Eqs. (A2.1)-(A2.18)). Length normalization between the starting (sacral plane) and the ending point (apex of thoracic kyphosis (curving of the spine posteriorly)) ensures that arc-length runs from 0 to *L*.

The curvilinear coordinate *S* (*X*(*S*)*,Y*(*S*)) represents a parametrization by the arc length along the spine curve while the inclination of the curve with respect to the *y*-axis is characterized by the winding angle ψ(*S*). Curvature *C* at arc length *S* is defined as the reciprocal value of the radius of the osculating circle. Positive curvature indicates a convex curve (lordosis) while a negative curvature indicates concave curvature (kyphosis). The curvature at any point of the spine curve can be determined as a ratio of change in the winding angle along the curve *S*

(1) *C* = dψ*/*d*S*.

The spine curve initiates at the lumbosacral joint with a sacral slope angle ψ_0_ (Fig. 1b) that is related to the pelvic incidence angle (PI) and the pelvic tilt angle (PT) (Fig. 1b),

(2) ψ_0_ = PI-PT,

where PI is defined as the angle between the line perpendicular to the sacral plate at its midpoint and the line connecting this point to the axis through the femoral heads [29], and PT is the orientation of the pelvis with respect to the thighbones and the rest of the body [30,31] (Fig. 1b).

We modeled the spine as an elastic beam with variable curvature in an unloaded configuration. The bending energy *W*_b_ was derived from the beam theory,

(3) *W*_b_ = *k*_b_/2 ∫ (*C*(*S*)-*C*_0_(*S*))^2^ d*S*,

where *k*_b_ is the bending stiffness and *C*_0_ is the inherent curvature. The inherent curvature is a measure of how much a given spine segment tends to bend or curve if not connected with other segments. The integration is performed along the distance from the origin of the coordinate system at the lumbosacral joint *S* = 0 to the apex of the thoracic kyphosis *S* = *L.* As the spine changes its curvature from lordosis to kyphosis, it is reasonable to assume that inherent curvature is arc-length dependent. In the first approximation, we assumed that the inherent curvature is a linear function of the arclength,

(4) *C*_0_(*S*) = ς_0_*S*,

where ς_0_ is a constant which we call the inherent curvature increment. For clarity, we define normalized quantities

(5) *s* = *S*/*L, x* = *X/L, y* = *Y/L,* and *c*(*s*) = *C*(*S*) *L*

and normalized arc length *s* ranges from 0 to 1. The dimensionless bending energy *w*_b_ = 2*W*_b_*L/k*_*b*_ now reads

(6) *w*_b_ = *∫* (*c*(*s*) *-* CI *s*)^*2*^*ds*.

where

(7) CI = ς_0_*L*^2^

is the dimensionless curvature increment. Integration is performed from *s* = 0 to *s* = 1. We assume that in equilibrium, the spinal curve has a shape that corresponds to the minimum of the bending energy (Eq. (6)).

In the model, the dimensionless bending energy (Eq. (6)) is minimized at constraint

(8) ∫ d*s* = 1.

The integration is performed from 0 to 1. The Euler-Lagrange method is used to state the variational problem. The Lagrange function L is constructed

(9) L(ψ, *y, s*) = (∂ψ/∂*s* – CI *s*)^2^ + λ(*s*)(∂*y*/∂*s* – cos ψ(*s*)) + λ_*L*_.

Here, λ(*s*) is the local Lagrange multiplier and λ_*L*_ is the global Lagrange multiplier. The extremales are subjected to the Euler-Lagrange equations

(10) ∂L/∂ψ - d/d*s*(∂L/∂(∂ψ/∂*s*)) = 0,

(11) ∂L/∂*y* - d/d*s*(∂L/d(∂*y*/∂*s*)) = 0,

(12) ∂L/∂λ = 0.

Inserting Eq. (9) into Eqs. (10), (11), (12) and performing the operations yields a system of differential equations

(13a) 2 (d^2^ψ(*s*)/d*s*^2^ - CI) - λ(*s*) sinψ(*s*) = 0,

(13b) dλ(*s*)/d*s* = 0,

(13c) d*y*/d*s* – cos ψ(*s*) = 0,

(13d) d*x*/d*s* – sin ψ(*s*) = 0.

The boundary conditions are

(14) ψ(0) = ψ_0_,

(15) ψ(1) = 0

and

(16) dψ/d*s* (0) = *c*_LS__,_Parameter *c*_LS_

is called the normalized lumbosacral curvature.

The system of equation (13) was solved numerically in Wolfram Mathematica (Wolfram Research, Inc., Mathematica, Version 11.2, Champaign, IL (2012)). The parameter ψ_0_ was the input into the model. The initial values of *x, y*, and λ are arbitrary and were taken to be 0. The energy minimum with respect to two model parameters (*c*_LS_ and CI) was sought. The results of the minimization procedure are the values of *c*_LS_ and CI corresponding to the shape with minimal energy, the shape of the spine with minimal energy *y*(*x*) (Fig. 1c), and the equilibrium normalized energy *w*_b_. For each spine, parameters ψ_0,_ *c*_L___S__, and CI characterize its equilibrium shape.

## 3 Materials and methods

### 3.1 Patients and normal subjects

**Fig. 2:**
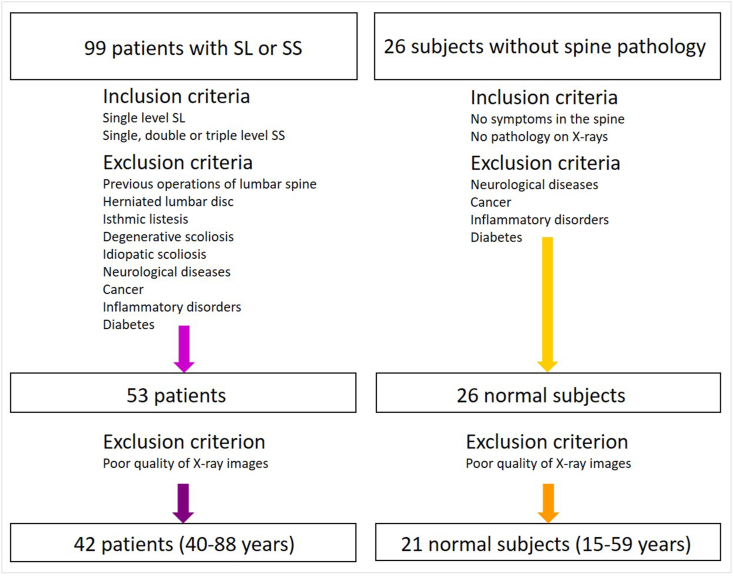
Scheme showing composition of the populations of patients and normal subjects.

The research included 99 patients with one-level SL with or without stenosis and patients with one-, two- or three-level SS, who were scheduled for decompression surgery due to SS or decompression and stabilization of vertebrae in SS with degenerative SL in the period from January 2019 to February 2020. After the exclusion of patients previously undergoing lumbar surgery, with a history of lumbar disc herniation, degenerative scoliosis, idiopathic scoliosis, neurologic disease, tumors or infections, and diabetes, the population of patients consisted of 53 patients. Another 11 patients were excluded due to insufficient quality of the X-ray images. Finally, the group of patients consisted of 42 persons (17 males and 25 females), their average age was 66 years with the range between 40 and 88 years. The control group consisted of initially 26 subjects without spine pathology in the records. After exclusion of 5 subjects due to insufficient quality of the X-ray images the final population of 21 normal subjects consisted of 11 males and 10 females. The average age of the normal subjects was 43 years and the age range was 15–59 years. The scheme of composing the populations of patients and normal subjects is shown in Fig. 2.

### 3.2 Assessment of geometrical parameters from radiographs

The sagittal spine radiographs of the patients and of the normal subjects were obtained from the archive of the Department of Orthopaedic Surgery, Ljubljana University Medical Centre. The radiographs were taken in the standing position. A mean sagittal shape of a radiograph was obtained by manually locating the centroids of vertebrae. Centroid labeling was chosen due to easier identification in some of the images of poorer quality. We assessed standard sagittal balance parameters: lumbar lordosis LL, pelvic incidence PI, pelvic tilt PT, sacral slope ψ_0_, and sagittal balance SB (Fig. 1b). Parameter ψ_0_ was the input into the model. The results of the minimization procedure were the equilibrium values of c_LS_ and CI, the equilibrium shape of the spine, and the equilibrium bending energy of the spine *w*_b_. Standard geometrical parameters LL, PI, PT, ψ_0,_ and the parameter of sagittal balance SB were assessed for all images (42 of patients and 21 of normal subjects).

The geometrical and biomechanical parameters were correlated between themselves and with the sagittal balance parameter SB [31]. SVA is the distance from the sacral line to the C7 plumb line and SFD is the distance between the hip axis line and the sacral line **(**Fig. 1a**).** This ratio is 0 when the C7 plumb line projects exactly on the posterior corner of the sacrum, and 1 when the C7 plumb line projects exactly on the bicoxofemoral axis. The ratio is negative when the C7 plumb line projects posteriorly to the sacrum and larger than 1 when the C7 plumb line projects from anterior to the femoral heads. Geometrical parameters of the assessed spines are given in the Supplementary material (Table 3).

### 3.3 Statistical methods

The collected data were initially subjected to descriptive statistical analysis to characterize the populations. Measures such as mean, standard deviation (SD), median, and range were calculated for continuous variables. Before further analysis, the normality of continuous variables was assessed using the Kruskal-Wallis test. QQ plots were utilized to assess the normality assumption for each parameter. The correspondences of the standard and the equilibrium parameters with the sagittal balance parameter SB were tested by using Pearson correlation coefficient. Two-tailed probability was calculated by using p-Value Calculator for Correlation Coefficients (Free Statistics Calculators, version 4.0, Daniel Soper, https://www.danielsoper.com/statcalc/calculator.aspx?id=44). Relationships were graphically represented using scatterplots with 95 % confidence intervals. Differences between groups were assessed using independent samples t-tests and statistical power analyses. The difference in means of variables by a group of patients was tested using the Welch Two Sample *t*-test test. To investigate interdependencies among variables, a linear regression model was employed. The association between the predictor geometric variable and model parameters, and the outcome variable of sagittal balance was analyzed. The statistical power of the differences between patients and normal groups was calculated by using HyLown Consulting online calculator (HyLown Consulting LLC, Atlanta, GA, USA, https://hylown.com/). Other statistical analyses were performed using R Statistical Software (v4.1.2; R Core Team 2021). Significance was set at α = 0.05.

### 3.4 Design of the study

By using a geometrical parameter ψ_0_ as an input to the model, we used the model presented in Section 2 to determine respective biomechanical parameters *c*_LS_ and CI for each spine. Parameters ψ_0,_ *c*_LS,_ and CI characterize its equilibrium shape. We assessed geometrical and biomechanical parameters for each shape in 42 patients diagnosed with SS or SL and 21 radiologically normal subjects for whom the spine was found to be without a record of disease. To better understand the biomechanical impact in spine pathology we validated the model by inter-relating the geometrical and biomechanical parameters. We were particularly interested in the performance of the geometrical parameters versus the biomechanical parameters *c*_LS_ and CI with an independent parameter of the sagittal balance SB. For validation, we used statistical methods described in Section 3.2.

## 4 Results

**Fig. 3:**
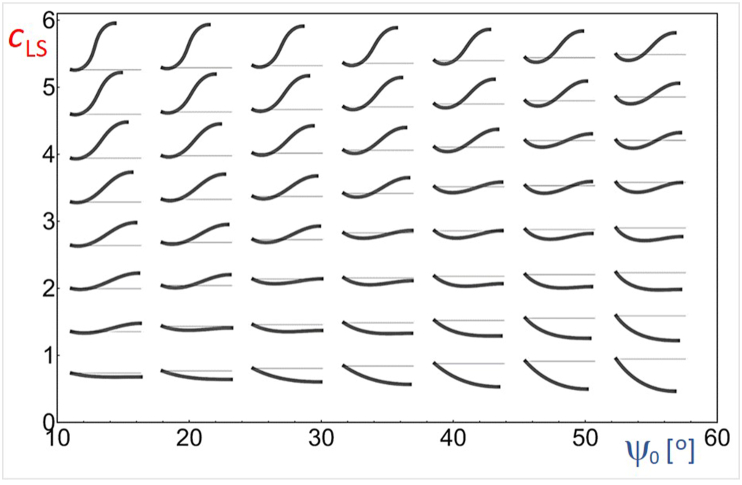
A (ψ_0_*, c*_LS_) phase diagram of the equilibrium shapes with minimal elastic energy (set of solutions of differential equation (13)).

Different shapes with minimal elastic energy corresponded to different parameters (ψ_0_, *c*_LS_)**:** from kyphosis observed at low angles ψ_0_ and high curvatures *c*_LS_, through flat back at low *c*_LS_ to hyperlordosis associated with large ψ_0_ and low *c*_LS._ As shown in the phase diagram (Fig. 3), the angle ψ_0_ itself does not fully predict the shape of lumbar spine curvature.

**Fig. 4:**
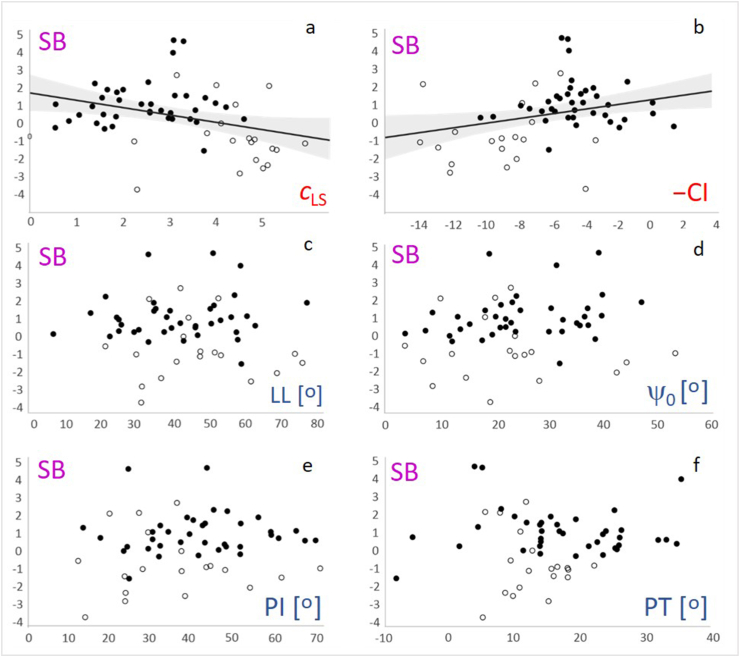
Interdependences between sagittal balance parameter SB and spine parameters: normalized lumbosacral curvature *c*_LS_ (a), normalized curvature increment CI (b), lumbar lordosis LL (c), sacral slope ψ_0_ (d), pelvic tilt PT (e) and pelvic incidence PI (f). The results of the patients with SS or SL are represented by full circles and the results of the normal subjects are represented by empty circles.

The model was validated on the populations of spines of patients and subjects with no record of disease. Raw data for all spines considered are given in Appendix 3 (Table 3). Interdependence between geometrical and biomechanical parameters and the sagittal balance SB was evaluated by calculating Pearson cross-correlations (Table 1) and more information is given in Fig. 4.

**Table 1:** The Pearson correlation coefficients and the corresponding probabilities (in parentheses) showing relationships between variables – geometrical parameters: lumbar lordosis (LL), pelvic incidence (PI), pelvic tilt (PT) and sacral slope ψ_0_, biomechanical parameters - normalized lumbosacral curvature *c*_LS_, and normalized curvature increment CI and sagittal balance SB.

All participants	PI	PT	ψ_0_	*c*_LS_	−CI	SB
LL	0.62 (<10^−4^)∗∗∗	0.01 (0.93)	0.82 (<10^−4^)∗∗∗	0.48 (10^−4^)∗∗∗	−0.26 (0.04)∗	−0.05 (0.70)
PI		0.67 (<10^−4^)∗∗∗	0.79 (<10^−4^)∗∗∗	−0.02 (0.88)	0.08 (0.54)	0.16 (0.23)
PT			0.08 (0.52)	−0.27 (0.03)∗	0.19 (0.14)	0.07 (0.62)
ψ_0_				0.2 (0.12)	−0.04 (0.75)	0.12 (0.35)
*c*_LS_					−0.87 (<10^−4^)∗∗∗	−0.34 (0.01)∗∗
−CI						0.33 (0.01)∗∗
Patients	PI	PT	ψ_0_	*c*_LS_	−CI	SB
LL	0.67 (<10^−4^)∗∗∗	0.07 (0.66)	0.86 (<10^−4^)∗∗∗	0.44 (4 × 10^−3^)∗∗	−0.24 (0.12)	0.10 (0.53)
PI		0.68 (<10^−4^)∗∗∗	0.72 (<10^−4^)∗∗∗	0.24 (0.12)	−0.14 (0.38)	0.13 (0.41)
PT			−0.01 (0.95)	−0.11 (0.49)	0.05 (0.75)	−0.05 (0.75)
ψ_0_				0.45 (3 × 10^−3^)∗∗	−0.26 (0.10)	0.23 (0.14)
*c*_LS_					−0.79 (<10^−4^)∗∗∗	0.06 (0.70)
−CI						0.05 (0.75)
Normal subjects	PI	PT	ψ_0_	*c*_LS_	−CI	SB
LL	0.79 (<10^−4^)∗∗∗	0.10 (0.67)	0.90 (<10^−4^)∗∗∗	0.49 (0.02)∗	0.05 (0.83)	−0.05 (0.83)
PI		0.63 (2 × 10^−3^)∗∗	0.90 (<10^−4^)∗∗∗	0.18 (0.43)	0.26 (0.25)	−0.36 (0.11)
PT			0.25 (0.27)	−0.13 (0.57)	0.23 (0.32)	−0.15 (0.52)
ψ_0_				0.20 (0.28)	0.29 (0.20)	−0.23 (0.32)
*c*_LS_					−0.80 (<10^−4^)∗∗∗	0.19 (0.40)
−CI						−0.27 (0.24)

Three asterisks denote strong correlation (p smaller or equal to 10^−4^), two asterisks denote moderate correlation (p smaller or equal to 0.01) one asterisk denotes weak correlation (p smaller than 0.05).

While strong correlations between some geometrical parameters and also between the two biomechanical parameters were revealed, only the model parameters *c*_LS_ and CI taking into account all participants (patients and normal subjects) showed a statistically significant correlation with the sagittal balance SB (Table 1, Fig. 4a and b). The correlations between geometrical parameters and SB, respectively, were not statistically significant (Table 1, Fig. 4c,d,e,f). Analyzing separately patients and normal subjects showed the same qualitative relations between geometrical parameters and between geometrical parameters and SB. However, the statistical significance of correlations that were weak in the group of all participants (between *c*_LS_ and SB, between CI and SB, between CI and LL, and between *c*_LS_ and PT were lost in separate groups due to smaller sample sizes (Table 1). However, the correlation between *c*_LS_ and ψ_0_ which was not exhibited in the group of all participants was revealed in the separate patients group (Table 1).

Average values of geometrical parameters (LL, PI, PT, ψ_0_), of the sagittal balance parameter SB and of the equilibrium biomechanical parameters *c*_LS_ and CI are given in Table 2. Statistically significant differences between the patients and normal subjects (p < 0.05) with sufficient statistical power (P > 0.8) were obtained in sagittal balance parameter SB and in biomechanical parameters *c*_LS_ and CI, but not in geometrical parameters LL, PI, PT, and ψ_0_ (Table 2).

**Table 2:** Average values and standard deviations of spine parameters lumbar lordosis (LL), pelvic incidence (PI), and sacral slope ψ_0_, of biomechanical parameters *c*_LS_ and CI and of sagittal balance parameter SB for all subjects considered, for patients and for normal subjects, the range of values for the respective groups, the difference between the patients and normal subjects, the probability reflecting the statistical significance of the difference p, and statistical power of the statistically significant difference P.

Parameter	Average All±SD	Average Normal±SD	Average Patients±SD	Range Normal	Range Patients	Difference Normal/Patients (%)	p	P
LL [^0^]	42.9 ± 15.7	47.9 ± 15.3	40.5 ± 15.4	21.4–76.1	6.9–77.3	18.3	0.076	
PI [^0^]	39.5 ± 15.4	34.3 ± 16.3	42.2 ± 14.4	8.7–72.0	13.5–70.9	18.6	0.056	
PT [^0^]	15.4 ± 9.04	11.9 ± 6.15	17.2 ± 9.8	−2.0 – 22.4	−7.3 – 35.5	30.6	0.029	0.42
ψ_0_ [^0^]	24.1 ± 11.4	22.7 ± 12.9	24.8 ± 10.6	3.7–53.8	3.8–47.6	8.5	0.493	
*c*_LS_	3.05 ± 1.52	4.53 ± 1.02	2.31 ± 1.14	2.2–6.2	−0.3 – 4.6	95.8	<0.001	1∗
−CI	5.56 ± 4.30	9.27 ± 3.04	3.86 ± 3.66	−13.8 – (−3.3)	−10.2 – 13.91	134.7	<0.001	1∗
SB	0.57 ± 1.67	−0.69 ± 1.74	1.19 ± 1.26	−3.6 – 2.8	−1.5 – 4.8	158.0	<0.001	1∗

Statistical significance of the difference between the groups was considered at p < 0.05 and sufficient statistical power was considered at P > 0.8. https://www.stat.ubc.ca/∼rollin/stats/ssize/n2.html. The difference in % was calculated as the difference divided by the average. Asterisk marks statistically significant differences with sufficient statistical power.

**Fig. 5:**
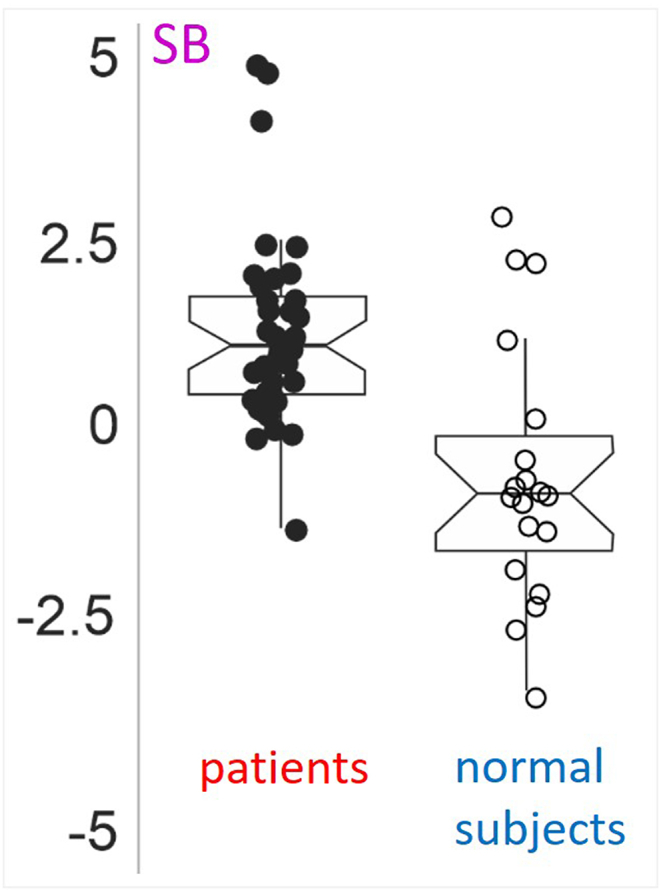
Boxplot of estimated sagittal balance (SB) for patients with SS or SL and normal subjects.

The sagittal balance SB differed significantly between the patients and the normal subjects groups (difference = 1.88, 95 % confidence interval [2.79, 0.96], t (27.49) = 4.19, p < 0.001; 95 %, confidence interval [2.44, 0.73]) (Fig. 5).

## 5 Discussion

We have described the state of the spine by modeling it as an elastic beam which in equilibrium corresponds to the minimum of the elastic (bending) energy. We stated and solved a variational problem to find the shape that yields minimal bending energy - by determining two model parameters (normalized curvature of the spine at the lumbosacral joint *c*_LS_ and normalized curvature increment reflecting the change of the inherent spine curvature increment CI). The input parameter of the model was the sacral slope angle ψ_0._ For given ψ_0,_ a set of differential equations was solved for different *c*_LS_ and CI (Fig. 3). The solution is not always feasible and some spine shapes do not describe the two-legged stance (Fig. 3), corresponding to cases where balance cannot be achieved. However, we could find the solutions that fitted all spines that were analyzed from radiographs.

Also, we have assessed geometrical parameters that were hitherto suggested for the description of the spine status. We found statistically significant, strong, positive correlation between PI and LL (r = 0.62, p < 10^−4^, Table 1), which is in agreement with previous reports (r = 0.75, p < 0.05 [30], r = 0.55, p < 0.05 [32], r = 0.53, p < 10^−4^ in patients subjected to operation due to adolescent idiopathic scoliosis (a condition in which the spine has an abnormal mediolateral curve) before operation and 0.67, p < 10^−4^ after the operation [33], r = 0.67 in patients with single level degenerative SL and 0.50 in normal subjects [12], between PI and PT (0.67, p < 10^−4^, Table 1) which is in agreement with previous report (r = 0.34, p < 0.05) [32], between PI and ψ_0_ (r = 0.79, p < 10^−4^, Table 1) which is in agreement with previous reports (r = 0.79 in patients with single level degenerative SL and 0.51 in normal subjects) [12] and (0.63, p < 0.05) [32], and between LL and ψ_0_ (r = 0.82, p < 10^−4^, Table 1) which is in agreement with previous reports r = 0.71, p < 0.05 [30], r = 0.76, p < 10^−4^ in patients subjected to operation due to adolescent idiopathic scoliosis before operation and 0.90, p < 10^−4^ after the operation [33], and (r = 0.83, p < 0.05) [32]. We found no statistical significance in PT - LL and PT - ψ_0_ correlations (r = 0.01, p = 0.93, Table 1) which agrees with the results of Imai et al. (2020) [32], however in contrast, Wu et al. (2020) [30] found statistically significant correlation between PT and LL in patients with modic changes (changes in the spinal endplate and sub-endplate bones observed on magnetic resonance images) (r = 0.69, p < 0.05).

We have validated the model parameters by correlating them with the parameter of sagittal balance SB [31]. The ratio is negative when the C7 plumb line projects posteriorly to the sacrum and larger than 1 when the C7 plumb line projects from anterior to the femoral heads. In our cohort of normal subjects, the average value of SB was −0.69 ± 1.74 which is within the same range as the previously reported result 0.9 ± 1 [31]. However, the average value of SB in our patients was considerably higher 1.19 ± 1.26 (Table 2, Fig. 5). In pathology, the compensatory mechanisms are not efficient enough to maintain the sagittal balance, as detailed in Ref. [31].

Instead of angles, the parameters of our model (*c*_LS_ and CI) express curvatures - i.e. changes in the winding angle along the spine curve. Curvature has previously been outlined as the relevant parameter [19,34], however, the work of Hay et al. (2009) [34] focused on the determination of the curvature along the spine from the images, and on the comparison of the shape with an average shape of normal spines. In contrast, we have derived the equilibrium spine curve theoretically by assuming that equilibrium corresponds to the minimum of the bending energy of the spine (from sacrum to apex of the thoracic kyphosis). The correlations of *c*_LS_ and CI with SB were statistically significant while the correlations of LL, PI, PT, and ψ_0_ with SB were not (Table 1). Thus, the biomechanical parameters *c*_LS_ and CI proved superior to geometrical parameters (LL, PI, PT and ψ_0_) in the correlation with the sagittal balance parameter SB (Table 1).

The spine, viewed from both physiological and biomechanical perspectives, represents a sophisticated mechanism optimized to execute diverse functions. The spine's interconnection becomes evident when even a localized alteration in mechanics significantly impacts functional parameters throughout the entire spinal column. Functional parameters are connected also with physical activity [35] and body position [36]. This study is based on the assumption that the spine is a unified entity and characterizes its performance through a global parameter. Thus, we introduced an original model of the thoracolumbar spinal column shape, built upon the principle of minimizing global energy.

The advantage of the proposed spinal model is its agreement with observed spinal shapes in an upright stance, achieved through the optimization of energy with respect to two parameters. Additionally, both parameters were found to be predictive concerning sagittal balance (Table 1, Table 2, Fig. 4a and b). The findings show the applicability of a simple yet comprehensive approach in forecasting spinal shape. However, this study limits its consideration of the spine up to the thoracolumbar region, where the angle relative to the sagittal axis reaches zero, and it operates solely in two dimensions, disregarding potential curvature in the mediolateral direction. Also, we employed a simple linear dependency of the inherent curvature on the position while the alternating sign of lumbar, thoracic, and cervical curvatures suggests the potential utility of a semi-empirical function that reflects these properties. These limitations might be addressed through further model development.

The model as presented does not explicitly consider discrete level deformation, however, this could be taken into account by upgrading it with additional boundaries and respective relevant boundary conditions. Furthermore, expanding the model to encompass three-dimensional space would enhance its applicability, although the principle of energy minimization remains universally applicable and could be extended to describe spinal curvature comprehensively. The model was here primarily designed and validated for the thoracolumbar spine but its physical principles are valid for other spinal segments as well. For instance, an analysis of the phase diagram could elucidate changes in cervical spine curvature associated with the use of mobile devices. The forward and downward bending of the head while utilizing handheld devices decreases the angle ψ_0_, leading to straightening or even reversing the natural cervical lordotic curve, akin to clinical observations [37]. Furthermore, the model could predict the change of the global equilibrium of the posture due to local changes caused by operation [3], in particular, if it involves grafts and fixation devices [38,39] and complements biomechanical analysis considering particular spine segments [7,40,41]. The model presented attempts to predict possible complications of the planned surgery and post-surgery rehabilitation [42]. Clinically, the potential use of the model is in predictions of global spine shape after medical interventions on specific vertebrae. Also, it would be of interest to connect the results of model predictions with clinical data to estimate the risk for spine disorders such as herniated discs (slipped inter-vertebrate discs), SS, and SL. By evolving the model into three dimensions, it becomes plausible to simulate additional clinical states, such as the progression of scoliosis.

We made a statistical analysis of the correlations between geometrical parameters, biomechanical parameters, and SB also in separate groups of patients and normal subjects. In general, the results in the separate groups were qualitatively similar to the combined group. As the sample sizes were smaller, correlations that were weaker already in the combined group (between *c*_LS_ and SB, between CI and SB, between CI and LL, and between *c*_LS_ and PT) have lost statistical significance in separate groups. There was one correlation that showed statistical significance only in a group of patients (between *c*_LS_ and ψ_0_). There was a considerable (cca 100 %) and statistically significant difference between patients and normal subjects in parameter *c*_LS_ while the difference in ψ_0_ was notably smaller (less than 10 %; Table 2). This contributed additionally to the scattering of points in the *c*_LS_ (ψ_0_) dependence of the combined group where a statistically significant correlation was not revealed.

It can be seen in Table 2 that SB and the biomechanical parameters *c*_LS_ and CI are subject to large standard deviations (even greater than the average values). Moreover, also the geometrical parameters showed standard deviations larger than about 20 % of the mean. Consequently, the respective values of parameters from patients and normal subjects were highly overlapping which indicates that more relevant parameters to validate the model should be sought. In the future, it would be of interest to expand statistical analysis by using additional methods such as Support Vector Machines and Principal Component Analysis to increase predictability.

## 6 Conclusions

We have constructed a global model predicting thoracolumbar spine curvature in the sagittal plane of the body based on the minimization of elastic energy of the spine. In the model, the spine shape is represented by two principal parameters: the normalized lumbosacral curvature *c*_LS_ and the normalized curvature increment CI. The input data into the model is the winding angle taken at the lumbosacral interface ψ_0_. We stated the variational problem taking into account the length constraint, for which the solution was sought numerically. In a certain range of parameters, the solution agreed with the general shape of the respective spine part in the two-legged stance. We validated the model on two populations: 42 patients with SS and LS and 21 normal subjects assuming that the same physical laws underlie their sagittal balance. We found a statistically significant correlation between the model parameters *c*_LS_ and −CI (Pearson coefficient −0.87 in the group of all participants, −0.79 in patients, and −0.8 in normal subjects) and statistically significant correlations between *c*_LS_ and SB and between −CI and SB (Pearson correlation coefficients −0.34 and 0.33, respectively, in the group of all participants). We found statistically significant differences between patients and normal subjects with sufficient statistical power (p < 10^−3^, P > 0.8) in both model parameters *c*_LS_ and CI and in the parameter SB. This indicates that the biomechanical parameters could serve as predictors of the SB and the clinical status of the spine. Future clinical studies are therefore highly warranted to validate and further develop the model. The potential of the model lies in its ability to link local changes or interventions to global changes in spinal posture.

## Funding

Authors acknowledge funding from Slovenian Agency for Research and Innovation (ARIS), grant numbers P2-0232 and P3-0388.

## Data availability

All the data are contained within the manuscript. Raw data is given in Appendix (Table 3).

## CRediT authorship contribution statement

**Špela Bračun:** Writing – review & editing, Writing – original draft, Visualization, Methodology, Investigation, Formal analysis, Data curation. **Anna Romolo:** Writing – review & editing, Writing – original draft, Visualization, Project administration, Investigation, Formal analysis, Data curation. **Veronika Rehakova:** Writing – review & editing, Methodology, Investigation, Formal analysis, Data curation. **Jure Leban:** Writing – review & editing, Visualization, Investigation, Formal analysis, Data curation. **Žan Pukšič:** Writing – review & editing, Investigation, Formal analysis, Data curation. **Rok Vengust:** Writing – review & editing, Validation, Supervision, Resources, Project administration, Methodology, Investigation, Funding acquisition, Conceptualization. **Matej Daniel:** Writing – review & editing, Writing – original draft, Visualization, Validation, Supervision, Software, Methodology, Investigation, Formal analysis, Data curation, Conceptualization. **Veronika Kralj-Iglič:** Writing – review & editing, Writing – original draft, Visualization, Validation, Supervision, Resources, Project administration, Methodology, Investigation, Funding acquisition, Formal analysis, Data curation, Conceptualization. **Mitja Drab:** Writing – review & editing, Writing – original draft, Visualization, Validation, Supervision, Software, Methodology, Investigation, Formal analysis, Data curation.

## Declaration of competing interest

The authors declare that they have no known competing financial interests or personal relationships that could have appeared to influence the work reported in this paper.

## Figures and Tables

**Table 3:** Raw data on geometrical parameters (Fig. 1) and data on biomechanical parameters of spines.

#	LL	PI	PT	ψ_0_	*c*_LS_	−CI	SB	Group	M	F	Age
1	50.53	52.39	14.49	37.60	3.35	−4.97	1.65	Patient		1	65
2	58.88	67.94	35.53	31.84	3.07	−4.89	4.08	Patient		1	55
3	22.63	23.52	11.77	12.01	1.43	−2.42	0.10	Patient		1	71
4	30.68	48.41	34.85	14.01	1.85	−2.73	0.47	Patient	1		65
5	59.13	24.88	−7.33	32.41	3.73	−6.10	−1.46	Patient		1	65
6	25.14	39.75	17.73	22.41	1.34	−2.55	1.05	Patient		1	71
7	53.38	59.94	23.75	33.01	4.21	−7.77	1.01	Patient	1		80
8	33.35	32.22	19.67	12.49	1.60	−1.88	−0.21	Patient	1		88
9	46.99	46.94	25.85	19.93	3.57	−6.29	0.16	Patient		1	53
10	42.20	17.78	−4.88	23.40	3.55	−7.24	0.84	Patient		1	77
11	35.37	43.61	12.28	30.83	3.11	−4.18	1.66	Patient		1	52
12	34.90	32.56	14.28	18.61	3.76	−5.63	1.53	Patient	1		66
13	45.25	39.85	17.15	22.58	−0.26	13.91	1.94	Patient		1	62
14	62.98	70.87	33.28	37.70	2.57	−6.51	0.70	Patient		1	67
15	43.11	41.97	23.77	18.04	0.55	1.37	−0.15	Patient		1	65
16	57.24	45.72	8.45	40.33	2.54	−4.74	2.42	Patient	1		51
17	51.52	40.70	19.67	21.53	1.87	−3.89	1.85	Patient		1	59
18	29.38	24.38	2.17	24.23	3.44	−4.93	0.34	Patient	1		70
19	32.14	32.32	10.54	22.05	1.03	−3.35		Patient		1	77
20	17.29	13.53	4.97	8.84	1.92	−5.44	1.42	Patient		1	52
21	24.54	30.65	17.20	13.51	0.56	0.11	1.17	Patient		1	57
22	25.16	32.52	26.17	7.54	2.97	−9.42	0.39	Patient		1	52
23	56.33	59.80	24.21	37.04	2.60	−4.05	1.18	Patient		1	66
24	38.43	34.51	14.54	20.54	2.39	−4.71	1.18	Patient	1		48
25	50.77	61.74	26.28	35.58	2.81	−5.93	0.82	Patient		1	79
26	46.43	68.23	32.06	36.16	3.02	−5.73	0.69	Patient	1		71
27	77.31	56.75	10.46	47.59	2.00	−4.82	2.00	Patient	1		54
28	39.37	42.97	16.85	25.12	1.55	−3.24	1.54	Patient	1		77
29	25.81	30.66	14.42	15.74	2.57	−4.55	0.76	Patient		1	74
30	51.30	44.06	4.43	39.66	3.08	−5.33	4.79	Patient		1	72
31	46.36	37.59	14.42	22.42	1.58	−3.44	0.60	Patient	1		69
32	6.94	29.58	25.59	3.78	0.84	−1.57	0.23	Patient	1		45
33	33.31	24.66	5.60	19.36	3.29	−4.96	4.72	Patient	1		69
34	34.83	39.16	15.91	23.64	1.66	−3.43	1.99	Patient	1		71
35	57.76	52.26	21.62	30.42	4.59	−10.18	0.34	Patient	1		80
36	39.75	43.55	22.90	21.40	1.06	0.13	0.57	Patient		1	77
37	21.45	49.10	25.48	24.39	1.39	−1.40	2.34	Patient	1		74
38	58.11	52.26	14.33	39.01	1.77	−4.46	−0.08	Patient		1	40
39	22.76	24.70	13.69	11.01	1.74	−3.09		Patient		1	79
40	37.70	48.80	14.27	32.85	3.05	−4.64	0.35	Patient		1	80
41	60.69	66.08	26.53	40.22	3.98	−6.13	1.24	Patient		1	71
42	12.03			12.51	0.47	−1.15		Patient	1		53
43	47.66	37.82	12.60	24.15	5.90	−13.79	−1.03	Normal	1		41
44	53.47	48.54	18.55	25.90	4.75	−9.50	−0.97	Normal		1	41
45	21.39	12.30	9.88	3.72	3.80	−11.70	−0.46	Normal	1		59
46	51.74	43.89	16.90	27.19	4.82	−8.91	−0.81	Normal	1		56
47	69.03	54.70	11.22	42.97	4.85	−8.02	−1.97	Normal		1	49
48	76.08	62.47	18.47	44.82	5.29	−8.90	−1.40	Normal	1		41
49	29.98	28.20	16.02	12.44	2.24	−3.30	−0.92	Normal		1	52
50	36.89	24.01	9.06	15.06	5.10	−11.90	−2.26	Normal	1		18
51	69.63	30.92	0.00	32.29	6.20	−11.70		Normal	1		15
52	44.49	29.54	11.35	18.58	4.41	−7.69	1.16	Normal	1		46
53	33.57	20.04	8.28	10.31	5.13	−13.61	2.21	Normal	1		34
54	42.25	36.66	12.18	23.37	3.15	−5.40	2.81	Normal	1		35
55	61.80	38.65	10.23	28.64	5.00	−8.60	−2.44	Normal		1	53
56	45.44	8.72	−2.00	10.97	5.40	−13.25		Normal		1	48
57	41.17	23.77	16.35	7.13	5.20	−12.70	−1.33	Normal		1	44
58	31.55	23.89	15.58	8.86	4.50	−12.00	−2.73	Normal	1		49
59	74.00	72.00	18.44	53.83	4.35	−7.60	−0.88	Normal		1	26
60	52.77	27.30	6.04	20.45	4.00	−6.90	2.25	Normal		1	45
61	31.36	14.03	5.68	19.55	2.30	−3.90	−3.63	Normal	1		46
62	43.09	37.78	13.13	24.12	4.10	−7.10	0.09	Normal		1	54
63	47.77	44.91	22.44	23.16	4.70	−8.10	−0.75	Normal		1	57

Legend: LL: lumbar lordosis, PI: pelvic incidence, PT: pelvic tilt, ψ_0_: sacral slope, *c*_LS_: normalized lumbosacral curvature, CI: normalized curvature increment, SB: sagittal balance, M: male, F: female. In some cases we were unable to assess some geometrical parameters from the images due to poor quality of images. However, we have retained in the analysis the parameters on the spine shape which could give useful information.

## Appendix 1 List of abbreviations, symbols and medical terms

### Abbreviations

SS: spinal stenosis
SL: spondylolisthesis

**Symbols**
*C*: curvature
*C*_0_: inherent curvature
CI: normalized curvature increment along the spine; CI = ς_0_*L*^2^
*c*: normalized curvature
*c*_LS_: dimensionless lumbosacral spinal curvature; *c*(*s*) = *C*(*S*) *L*
d*s,* d*x,* d*y*: infinitesimal arclength and distances, respectively
*k*_b_: bending stiffness
*L*: length of the spine between the sacral plane and apex of thoracic kyphosis
L: Lagrange function
LL: lumbar lordosis
λ(*s*): local Lagrange multiplier and
λ_*L*_: global Lagrange multiplier
p: probability
P: statistical power
PI: pelvic incidence
PT: pelvic tilt
ψ: inclination of the spine curve with respect to the vertical
ψ_0_: sacral slope angle
SVA: sagittal vertical axis
SFD: sacrum-bicoxofemoral distance
SB: sagittal balance; SB = SVA/SFD
*S*: arc length along the spine curve
*s*: normalized arclength along the spine curve; *s* = *S/L*
*W*_b_: bending energy
*w*_b_: dimensionless bending energy; *w*_b_ = 2*W*_b_*L/k*_*b*_
ς_0_: inherent curvature increment

**Medical terms**
cervical spine: spine pertaining to the first 7 vertebrae
decompression surgery: a type of surgery used to treat compressed nerves
degenerative scoliosis: a condition in which the spine has an abnormal medio-lateral curve and changes for the worse over time
disc herniation: slipped disc between the vertebrae
idiopathic: arising spontaneously or by unknown causes
spondylolisthesis/listhesis: displacement of the cranially located vertebra over the caudal one
kyphosis: curving of the spine posteriorly
lordosis: curving of the spine anteriorly
lumbar spine: spine pertaining to 5 vertebrae above the sacrum
modic changes: changes in the spinal endplate and sub-endplate bones observed on magnetic resonance images
para-spinal muscles: action muscles of the back
pedicle: cylinder-shaped projection of hard bone that sticks out from the back part of the vertebral body
sagittal balance: physiological alignment of the spine in the most efficient manner
sagittal plane of the body: vertical plane which passes through the body longitudinally
scoliosis: a condition in which the spine has an abnormal medio-lateral curve
spinal stenosis: diminished cross-sectional area of the spinal canal and/or intervertebral foramina
thoracic spine: spine pertaining to the 12 vertebrae above the lumbar spine and below the cervical spine

### Appendix A2 Derivation of model equations

In this Appendix we show step by step how to derive the system of differential equation (13).

We consider the Lagrange function L (Eq. (9))

(A2.1) L = (∂ψ/∂*s* – CI *s*)^2^ + λ(*s*)(d*y*/d*s* – cos ψ(*s*)) + λ_*L*_.

with λ(s) the local Lagrange multiplier and λ_*L*_ is the global Lagrange multiplier, and the Euler-Lagrange equations (Eqs. (10), (11), (12))

(A2.2) ∂L/∂ψ - d/d*s*(∂L/∂(∂ψ/d*s*)) = 0,

(A2.3) ∂L/∂*y* - d/d*s*(∂L/∂(∂*y*/∂*s*)) = 0,

(A2.4) ∂L/∂λ = 0.

The derivations

(A2.5) ∂L/∂ψ = λ(*s*) sin ψ(*s*),

(A2.6) ∂L/∂(∂ψ/∂*s*) = 2(∂ψ/∂*s* – CI *s*),

(A2.7) d/d*s*(∂L/∂(∂ψ/∂*s*)) = 2 (∂^2^ψ(s)/∂s^2^ - CI),

are inserted into (A2.2). After rearrangement we obtain Eq. (13a)

(A2.8) 2 (d^2^ψ(s)/ds^2^ - CI) - λ(*s*) sin ψ(*s*) = 0

The derivations

(A2.9) ∂L/∂*y* = 0,

(A2.10) ∂L/∂(∂*y*/∂*s*) = λ(*s*),

(A2.11) d/d*s*(∂L/∂ (∂*y*/∂*s*)) = dλ/d*s*,

are inserted into (A2.3). We obtain

(A2.12) dλ/d*s* = 0.

Deriving L with respect to λ gives

(A2.13) ∂L/∂λ = d*y*/d*s* – cos ψ(*s*)

to yield by considering Eq. (A2.4) the constraint (13c)

(A2.14) dy/d*s* – cos ψ(*s*) = 0.

Constraints (13c), (13d):

(A2.15) d*x*/d*s* – sin ψ(*s*) = 0.

derive from the choice of the parametrization (Fig. 1).

The boundary conditions

(A2.16) ψ(0) = ψ_0_,

and

(A2.17) dψ/d*s* (0) = *c*_LS._

follow from the morphology of the spine showing that the spinal curve starts at the interface between the sacrum and the spine with a nonzero angle and curvature. The condition

(A2.18) ψ(1) = 0

presents the choice of the end of the curve at the apex of thoracic kyphosis.
