# Supplementary material for: Correlation between sagittal balance and thoracolumbar elastic energy parameters in 42 spines subject to spondylolisthesis or spinal stenosis and 21 normal spines
Source: Heliyon. 2024 Sep 26;10(19):e38469. doi: 10.1016/j.heliyon.2024.e38469 (PMC11489354; doi:10.1016/j.heliyon.2024.e38469)
Supplement: Multimedia component 1 [file mmc1.docx]

# Appendix A3 Data

# Table 3. Raw data on geometrical parameters (Fig. 1) and data on biomechanical parameters of spines

| # | LL | PI | PT | ψ_0_ | *c*_LS_ | CI | SB | Group | M | F | Age |
| --- | --- | --- | --- | --- | --- | --- | --- | --- | --- | --- | --- |
| 1 | 50.53 | 52.39 | 14.49 | 37.60 | 3.35 | -4.97 | 1.65 | Patient |  | 1 | 65 |
| 2 | 58.88 | 67.94 | 35.53 | 31.84 | 3.07 | -4.89 | 4.08 | Patient |  | 1 | 55 |
| 3 | 22.63 | 23.52 | 11.77 | 12.01 | 1.43 | -2.42 | 0.10 | Patient |  | 1 | 71 |
| 4 | 30.68 | 48.41 | 34.85 | 14.01 | 1.85 | -2.73 | 0.47 | Patient | 1 |  | 65 |
| 5 | 59.13 | 24.88 | -7.33 | 32.41 | 3.73 | -6.10 | -1.46 | Patient |  | 1 | 65 |
| 6 | 25.14 | 39.75 | 17.73 | 22.41 | 1.34 | -2.55 | 1.05 | Patient |  | 1 | 71 |
| 7 | 53.38 | 59.94 | 23.75 | 33.01 | 4.21 | -7.77 | 1.01 | Patient | 1 |  | 80 |
| 8 | 33.35 | 32.22 | 19.67 | 12.49 | 1.60 | -1.88 | -0.21 | Patient | 1 |  | 88 |
| 9 | 46.99 | 46.94 | 25.85 | 19.93 | 3.57 | -6.29 | 0.16 | Patient |  | 1 | 53 |
| 10 | 42.20 | 17.78 | -4.88 | 23.40 | 3.55 | -7.24 | 0.84 | Patient |  | 1 | 77 |
| 11 | 35.37 | 43.61 | 12.28 | 30.83 | 3.11 | -4.18 | 1.66 | Patient |  | 1 | 52 |
| 12 | 34.90 | 32.56 | 14.28 | 18.61 | 3.76 | -5.63 | 1.53 | Patient | 1 |  | 66 |
| 13 | 45.25 | 39.85 | 17.15 | 22.58 | -0.26 | 13.91 | 1.94 | Patient |  | 1 | 62 |
| 14 | 62.98 | 70.87 | 33.28 | 37.70 | 2.57 | -6.51 | 0.70 | Patient |  | 1 | 67 |
| 15 | 43.11 | 41.97 | 23.77 | 18.04 | 0.55 | 1.37 | -0.15 | Patient |  | 1 | 65 |
| 16 | 57.24 | 45.72 | 8.45 | 40.33 | 2.54 | -4.74 | 2.42 | Patient | 1 |  | 51 |
| 17 | 51.52 | 40.70 | 19.67 | 21.53 | 1.87 | -3.89 | 1.85 | Patient |  | 1 | 59 |
| 18 | 29.38 | 24.38 | 2.17 | 24.23 | 3.44 | -4.93 | 0.34 | Patient | 1 |  | 70 |
| 19 | 32.14 | 32.32 | 10.54 | 22.05 | 1.03 | -3.35 |  | Patient |  | 1 | 77 |
| 20 | 17.29 | 13.53 | 4.97 | 8.84 | 1.92 | -5.44 | 1.42 | Patient |  | 1 | 52 |
| 21 | 24.54 | 30.65 | 17.20 | 13.51 | 0.56 | 0.11 | 1.17 | Patient |  | 1 | 57 |
| 22 | 25.16 | 32.52 | 26.17 | 7.54 | 2.97 | -9.42 | 0.39 | Patient |  | 1 | 52 |
| 23 | 56.33 | 59.80 | 24.21 | 37.04 | 2.60 | -4.05 | 1.18 | Patient |  | 1 | 66 |
| 24 | 38.43 | 34.51 | 14.54 | 20.54 | 2.39 | -4.71 | 1.18 | Patient | 1 |  | 48 |
| 25 | 50.77 | 61.74 | 26.28 | 35.58 | 2.81 | -5.93 | 0.82 | Patient |  | 1 | 79 |
| 26 | 46.43 | 68.23 | 32.06 | 36.16 | 3.02 | -5.73 | 0.69 | Patient | 1 |  | 71 |
| 27 | 77.31 | 56.75 | 10.46 | 47.59 | 2.00 | -4.82 | 2.00 | Patient | 1 |  | 54 |
| 28 | 39.37 | 42.97 | 16.85 | 25.12 | 1.55 | -3.24 | 1.54 | Patient | 1 |  | 77 |
| 29 | 25.81 | 30.66 | 14.42 | 15.74 | 2.57 | -4.55 | 0.76 | Patient |  | 1 | 74 |
| 30 | 51.30 | 44.06 | 4.43 | 39.66 | 3.08 | -5.33 | 4.79 | Patient |  | 1 | 72 |
| 31 | 46.36 | 37.59 | 14.42 | 22.42 | 1.58 | -3.44 | 0.60 | Patient | 1 |  | 69 |
| 32 | 6.94 | 29.58 | 25.59 | 3.78 | 0.84 | -1.57 | 0.23 | Patient | 1 |  | 45 |
| 33 | 33.31 | 24.66 | 5.60 | 19.36 | 3.29 | -4.96 | 4.72 | Patient | 1 |  | 69 |
| 34 | 34.83 | 39.16 | 15.91 | 23.64 | 1.66 | -3.43 | 1.99 | Patient | 1 |  | 71 |
| 35 | 57.76 | 52.26 | 21.62 | 30.42 | 4.59 | -10.18 | 0.34 | Patient | 1 |  | 80 |
| 36 | 39.75 | 43.55 | 22.90 | 21.40 | 1.06 | 0.13 | 0.57 | Patient |  | 1 | 77 |
| 37 | 21.45 | 49.10 | 25.48 | 24.39 | 1.39 | -1.40 | 2.34 | Patient | 1 |  | 74 |
| 38 | 58.11 | 52.26 | 14.33 | 39.01 | 1.77 | -4.46 | -0.08 | Patient |  | 1 | 40 |
| 39 | 22.76 | 24.70 | 13.69 | 11.01 | 1.74 | -3.09 |  | Patient |  | 1 | 79 |
| 40 | 37.70 | 48.80 | 14.27 | 32.85 | 3.05 | -4.64 | 0.35 | Patient |  | 1 | 80 |
| 41 | 60.69 | 66.08 | 26.53 | 40.22 | 3.98 | -6.13 | 1.24 | Patient |  | 1 | 71 |
| 42 | 12.03 |  |  | 12.51 | 0.47 | -1.15 |  | Patient | 1 |  | 53 |
| 43 | 47.66 | 37.82 | 12.60 | 24.15 | 5.90 | -13.79 | -1.03 | Normal | 1 |  | 41 |
| 44 | 53.47 | 48.54 | 18.55 | 25.90 | 4.75 | -9.50 | -0.97 | Normal |  | 1 | 41 |
| 45 | 21.39 | 12.30 | 9.88 | 3.72 | 3.80 | -11.70 | -0.46 | Normal | 1 |  | 59 |
| 46 | 51.74 | 43.89 | 16.90 | 27.19 | 4.82 | -8.91 | -0.81 | Normal | 1 |  | 56 |
| 47 | 69.03 | 54.70 | 11.22 | 42.97 | 4.85 | -8.02 | -1.97 | Normal |  | 1 | 49 |
| 48 | 76.08 | 62.47 | 18.47 | 44.82 | 5.29 | -8.90 | -1.40 | Normal | 1 |  | 41 |
| 49 | 29.98 | 28.20 | 16.02 | 12.44 | 2.24 | -3.30 | -0.92 | Normal |  | 1 | 52 |
| 50 | 36.89 | 24.01 | 9.06 | 15.06 | 5.10 | -11.90 | -2.26 | Normal | 1 |  | 18 |
| 51 | 69.63 | 30.92 | 0.00 | 32.29 | 6.20 | -11.70 |  | Normal | 1 |  | 15 |
| 52 | 44.49 | 29.54 | 11.35 | 18.58 | 4.41 | -7.69 | 1.16 | Normal | 1 |  | 46 |
| 53 | 33.57 | 20.04 | 8.28 | 10.31 | 5.13 | -13.61 | 2.21 | Normal | 1 |  | 34 |
| 54 | 42.25 | 36.66 | 12.18 | 23.37 | 3.15 | -5.40 | 2.81 | Normal | 1 |  | 35 |
| 55 | 61.80 | 38.65 | 10.23 | 28.64 | 5.00 | -8.60 | -2.44 | Normal |  | 1 | 53 |
| 56 | 45.44 | 8.72 | -2.00 | 10.97 | 5.40 | -13.25 |  | Normal |  | 1 | 48 |
| 57 | 41.17 | 23.77 | 16.35 | 7.13 | 5.20 | -12.70 | -1.33 | Normal |  | 1 | 44 |
| 58 | 31.55 | 23.89 | 15.58 | 8.86 | 4.50 | -12.00 | -2.73 | Normal | 1 |  | 49 |
| 59 | 74.00 | 72.00 | 18.44 | 53.83 | 4.35 | -7.60 | -0.88 | Normal |  | 1 | 26 |
| 60 | 52.77 | 27.30 | 6.04 | 20.45 | 4.00 | -6.90 | 2.25 | Normal |  | 1 | 45 |
| 61 | 31.36 | 14.03 | 5.68 | 19.55 | 2.30 | -3.90 | -3.63 | Normal | 1 |  | 46 |
| 62 | 43.09 | 37.78 | 13.13 | 24.12 | 4.10 | -7.10 | 0.09 | Normal |  | 1 | 54 |
| 63 | 47.77 | 44.91 | 22.44 | 23.16 | 4.70 | -8.10 | -0.75 | Normal |  | 1 | 57 |

Legend: LL: lumbar lordosis, PI: pelvic incidence, PT: pelvic tilt, ψ_0_: sacral slope, *c*_LS_: normalized lumbosacral curvature, CI: normalized curvature increment, SB: sagittal balance, M: male, F: female. In some cases we were unable to assess some geometrical parameters from the images due to poor quality of images. However, we have retained in the analysis the parameters on the spine shape which could give useful information.
